# Case Report: A novel *FGFR1* fusion in acute B-lymphoblastic leukemia identified by RNA sequencing

**DOI:** 10.3389/fonc.2023.1276695

**Published:** 2023-11-01

**Authors:** Zhibo Zhang, Yiyan Zhu, Zheng Wang, Zhao Zeng, Lijun Wen, Ling Zhang, Suning Chen

**Affiliations:** ^1^ Jiangsu Institute of Hematology, Key Laboratory of Thrombosis and Hemostasis of Ministry of Health, the First Affiliated Hospital of Soochow University, Soochow University, Suzhou, China; ^2^ Institute of Blood and Marrow Transplantation, Collaborative Innovation Center of Hematology, Soochow University, Suzhou, China; ^3^ Suzhou Jsuniwell Medical Laboratory, Suzhou, China

**Keywords:** acute lymphoblastic leukemia, Kif5B, FGFR1, rearrangement, 8p11 myeloproliferative

## Abstract

8p11 myeloproliferative syndrome is a rare hematological malignancy with aggressive course caused by the various translocation of *FGFR1*. In this study, a novel *FGFR1* fusion was identified by RNA sequencing in a 28-year-old male patient with acute B-lymphoblastic leukemia. The patient harbors an in-frame fusion between *KIF5B* exon 15 and *FGFR1* exon 10. The *FGFR1* fusion and its protein expression was validated by Sanger sequencing and Western blot. Meanwhile, cytogenetic analysis reported a normal karyotype and targeted DNA sequencing identified no driver mutations, respectively. Despite he achieved complete remission after induction regimen, a relapse occurred and he became refractory to chemotherapy, and salvage haploidentical hematopoietic stem cell transplantation failed to control the progressive disease. In conclusion, we present the first case of *KIF5B-FGFR1* fusion in hematological malignancy. These findings extend the spectrum of translocation in 8p11 myeloproliferative syndrome, and demonstrate the great prospect of RNA sequencing in clinical practice again.

## Introduction

Myeloid/lymphoid neoplasms with eosinophilia and tyrosine kinase gene fusions (MLN-TK) is a special category of hematologic malignancy driven by rearrangements of a subset of tyrosine kinase genes (1, 2). These rearrangements lead to fusion proteins in which the kinase domain is constitutively activated, with resulting oncogenic properties. 8p11 myeloproliferative syndrome (EMS) is a specific subtype of MLN-TK caused by a translocation involving *FGFR1* gene. Seventeen *FGFR1* fusions has been reported in EMS so far, of which the most common are *ZNF198-FGFR1*, *BCR-FGFR1* and *CEP110-FGFR1 (* 3). Most EMS patients are diagnosed with CML (chronic myeloid leukemia), aCML (atypical chronic myeloid leukemia), T-LBL (T-cell lymphoblastic lymphoma), or AML (acute myeloid leukemia). With aggressive clinical course and rapid progression, most EMS patients are resistant to traditional chemotherapeutic agents and most tyrosine kinase inhibitors (TKIs). Herein, we describe an in-frame *KIF5B-FGFR1* fusion gene firstly identified by RNA sequencing in a patient with acute B- lymphoblastic leukemia (B-ALL).

## Case description

**Figure 1:**
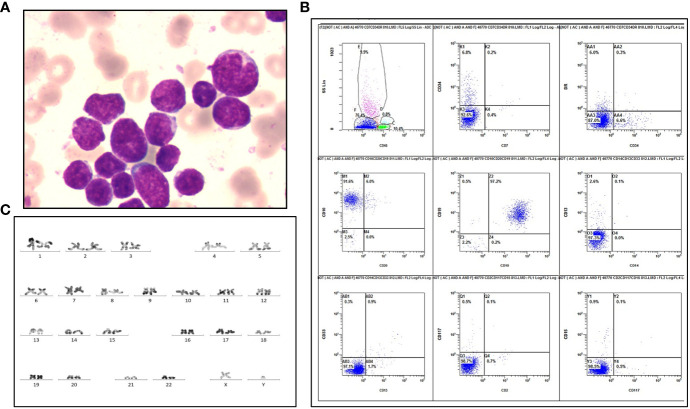
Laboratory examinations of the patient diagnosed as B-ALL. **(A)** Wright-Giemsa staining of blasts at diagnosis. **(B)** Flow cytometric immunophenotyping reveals a large CD45-negative blast population positive for CD10 and CD19, and negative for HLA-DR, CD2, CD7, CD13, CD14, CD15, CD20, CD33, CD34 and CD117. **(C)** R-band karyotyping shows 46, XY [20] in this patient.

A 28-year-old man was referred to our hospital, complaining of petechia, abdominal distension and hemosputum in July 2017. Sternum tenderness, lymphadenopathy of left neck and splenomegaly were found by physical examination. Complete blood count revealed that leukocytosis (125.55×10^9^/L), anemia (86 g/L) and thrombocytopenia (8 ×10^9^/L). At the same time, peripheral blood smear showed 67% blasts, 1% eosinophils, 5% neutrophils and 26% lymphocytes. Bone marrow (BM) aspirates revealed hypercellular with 64% blasts (**Figure 1A**). Immunophenotypic analysis showed a large subset of (76.4%) leukemic cells positive for CD10 and CD19 (**Figure 1B**). Cytogenetic analysis of the BM showed a normal karyotype: 46, XY (20/20 cells) (**Figure 1C**). Meanwhile, a fluorescence *in situ* hybridization (FISH) test for the detection of *BCR-ABL1* rearrangement and a targeted next-generation DNA sequencing using a panel of 172 genes (**Table S1**) were negative. Besides, a negative result was detected by a multiplex reverse transcription-polymerase chain reaction (RT-PCR) analysis for 43 acute leukemia-related fusion genes.

**Figure 2:**
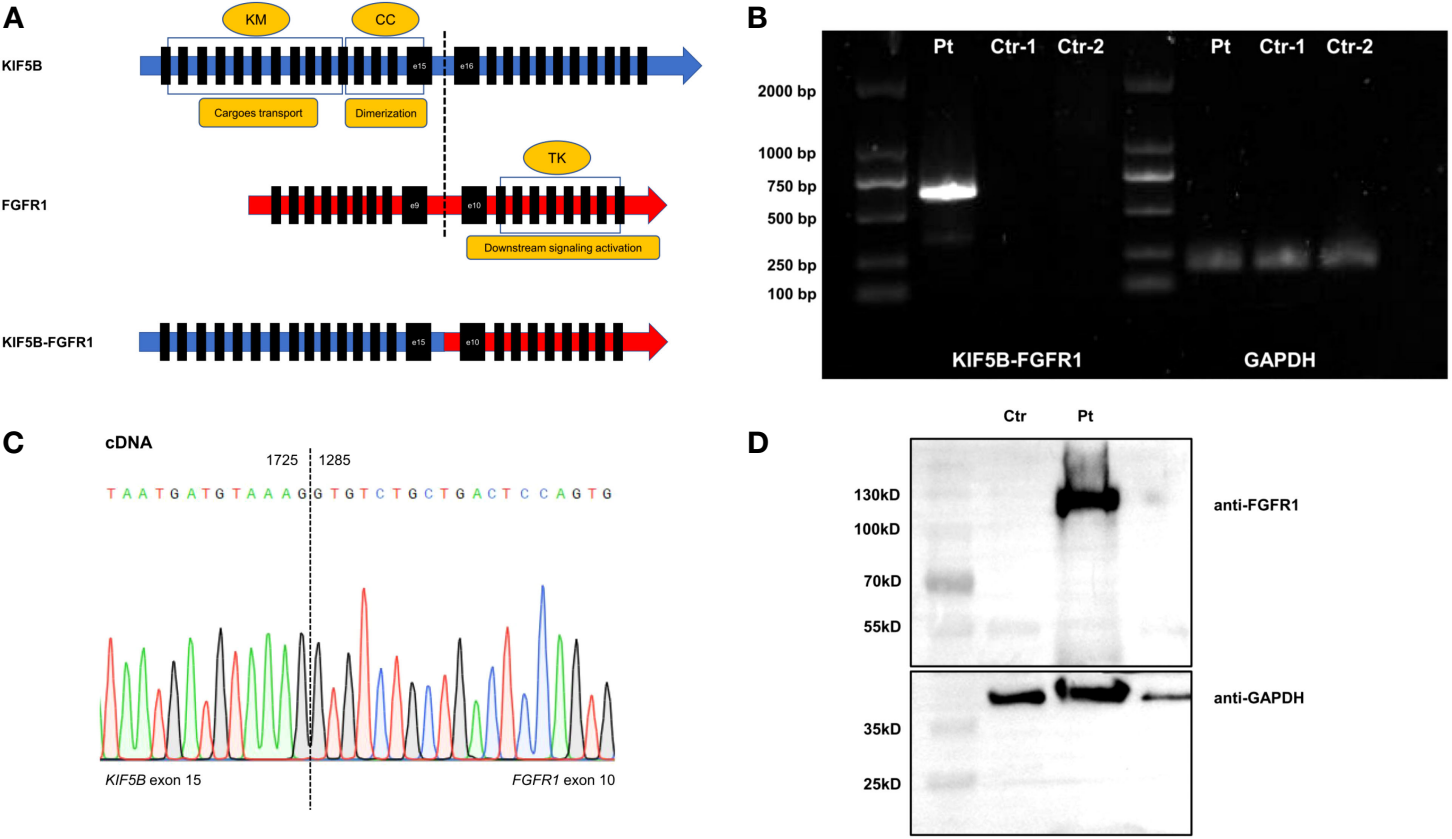
**(A)** The structure diagram of wildtype KIF5B, FGFR1, and KIF5B-FGFR1 fusion transcript. **(B)** Electrophoresis of RT-PCR products. Showing the positive KIF5B-FGFR1 fusion transcript in the patient’s bone marrow sample. Pt was the diagnostic sample. Ctr-1 and Ctr-2 were the negative control. **(C)** Sanger sequencing of the PCR product confirmed the fusion between exon 15 of the KIF5B gene and exon 10 of the FGFR1 gene. **(D)** Western blot analysis of fusion protein. Detecting one protein containing FGFR1 with a size between 100kD and 130 kD in the patient’s bone marrow sample. Pt was the diagnostic sample. Ctr was the negative control. KM, kinesin motor; CC, coiled coin; TK, tyrosine kinase.

However, RNA sequencing revealed a fusion involving exon1-15 of *KIF5B* (NM_004521.3) and exon10-18 of *FGFR1* (NM_023110.3), which mapped to chromosome 10p11 and 8p11, respectively (**Figure 2A**). The *KIF5B-FGFR1* mRNA was reverse-transcribed into cDNA using master mix for RT- PCR (Takara, Japan), and sequential PCR was performed using the following primers: *KIF5B* F: 5’-CAGGAGGAGCTTTTGGCATCT-3’, and *FGFR1* R: 5’-TGCGTCCGACTTCAACATCT-3’, which is the same as described previously (4). As a result, a 616-bp product was specifically amplified only from the patient’s cDNA (**Figure 2B**), which confirmed the presence of this fusion gene. Moreover, Sanger sequencing analysis of the fusion transcript revealed that *KIF5B* exon 15 was fused in-frame to *FGFR1* exon 10 (**Figure 2C**). Western blot was performed using the BM mononuclear lysates from the patient with a rabbit anti-FGFR1 antibody (Cell Signaling Technology, USA). As shown in **Figure 2D**, a protein band corresponding to the KIF5B fused FGFR1 was detected, which demonstrated the *KIF5B-FGFR1* fusion gene can lead to the formation of fusion protein.

According to above tests, the patient was diagnosed as B-ALL with rearrangement of *FGFR1*, which is a subtype of BCR-ABL1 like ALL. After supporting treatment, he received a course of induction chemotherapy using IVP regimen (idarubicin, vindesine and dexamethasone) and achieved complete remission (CR) with a detectable minimal residual disease (MRD), which was consistent with previous reports that *BCR-ABL1* like ALL correlated with MRD persistence. Consolidated with 2 courses of hyper-CVAD regimen (cyclophosphamide, vincristine, doxorubicin, and dexamethasone), the patient relapsed and was treated by a course of FLAG-IDA regimen (fludarabine, cytarabine, idarubicin, and granulocyte colony-stimulating factor). Without any improvement, he underwent haploidentical hematopoietic stem cell transplantation (HSCT) from his brother and achieved CR. However, the MRD become positive two months after transplantation and donor lymphocyte infusion was administered. Unfortunately, an early relapse finally occurred about one month later with pancytopenia, decreased short tandem repeats chimerism (86.5%) and 12% blasts detected in BM smears. Immunosuppressants were stopped and MOpAD regimen (methotrexate, vincristine, asparaginase, and dexamethasone) combined with DLI was used to control the disease, but he failed to achieve remission. Then the patient transferred to another hospital for economic reasons and died of progressive disease five months later.

## Discussion

B-ALL with *FGFR1* rearrangements belongs to *BCR-ABL1* like ALL, which is a poor-risk subset and shares a similar gene expression profile to that of ALL with *BCR-ABL1* (5, 6). In fact, there is some overlap between the *BCR-ABL1* like ALL and MLN-TK diagnostic criterion, and some Ph-like ALL cases could also be diagnosed as MLN-TK, for MLN-TK include a broad range of histologic types and have complex clinical manifestations. Although the latest International Consensus Classification (ICC) of myeloid neoplasms and acute leukemias distinguishes *BCR-ABL1* like ALL from MLN-TK presenting as B-ALL by identifying involvement of TK gene fusion in the background myeloid cells in addition to o lymphoblasts (7), the 5^th^ edition of the World Health Organization (WHO) Classification of hematolymphoid tumors does not emphasize the difference between these two groups and holds the opinion that the diagnosis of MLN-TK supersedes other myeloid and lymphoid types from a diagnostic hierarchy standpoint (1). The clinical characteristics (young male, absence of eosinophilia, no MPN history, poor outcome) of our case support a diagnosis of *BCR-ABL1* like ALL rather than typical MLN-TK according to ICC. However, we preferred a diagnosis of EMS (MLN-TK) according to the 5^th^ WHO classification, because it could better specify the underlying molecular genetic aberrations of such cases, considering *FGFR1* rearrangements are quite uncommon in *BCR-ABL1* like ALL (5, 8–11).

EMS is a rare subtype of hematologic neoplasms with fewer than 120 reported cases worldwide (3). The disease could occur at any age with protean and variable clinical manifestations, which makes it easy to ignore or misdiagnose as other hematologic malignancies. Apart from a male predominance, eosinophilia and monocytosis were noted in a substantial proportion of patients with EMS (12). According to previous literature, *ZNF198* (also called *ZMYM2*), located 13q11-12, is the most frequent gene partner of FGFR1 in EMS. Most patients with *ZNF198-FGFR1* are diagnosed with T-LBL/T-lymphoma, with the presence of eosinophilia and lymphadenopathy (13). In addition, the t(8;22) (p11;q11), which generates *BCR-FGFR1*, is the second common translocation in patients with EMS. Patients with *BCR-FGFR1* have clinical manifestations resembling CML and tend to develop B-ALL or lymphoma presenting with basophilia (14). Another common translocation in EMS is t(8;9)(p11-12;q32-34), which results in *CEP110-FGFR1* fusion gene. Of note, patients with *CEP110-FGFR1* often present with both eosinophilia and monocytosis (15). Besides, lymphadenopathy, purpura and skin lesions are also common symptoms at the time of diagnosis. Meanwhile, the initial diagnosis of patients with *CEP110-FGFR1* were diverse, including AML, T-LBL, and aCML. Besides, other recurrent fusion genes identified in EMS are *FOP1-FGFR1*, *HERVK-FGFR1*, *NUP98-FGFR1*, *FOP2-FGFR1*, *TIF1-FGFR1*, *MYO18A-FGFR1*, *TPR-FGFR1* and *NUP358-FGFR1 (* 3). It should be noted that G-banding karyotype analysis of our case failed to identify translocation between chromosome 10p11 and 8p11 at diagnosis. To better identify the patients with balanced reciprocal translocations, it is necessary to detect FGFR1 rearrangements by a combination of karyotype analysis, FISH and next-generation sequencing of molecular genetic techniques.

Aside from complex clinical presentation and molecular aberrations, most EMS patients are resistant to traditional treatment and thus have a very unfavorable prognosis. Previous studies showed that the CR rate and 1-year overall survival of EMS patients was merely 27% and 43.1%, respectively (12, 16). At present, allogeneic HSCT is the only method to improve the survival of EMS patients (17). Although non-selective TKIs such as imatinib, nilotinib, and dasatinib have showed limited efficacy on MLN with FGFR1 rearrangements (18), pemigatinib, a selective and potent inhibitor of FGFR1-3, has been approved for relapsed or refractory MLN with FGFR1 rearrangements due to promising results in the FIGHT-203 (NCT03011372) study (19), which may offer a long-term treatment option for EMS patients. Additionally, another selective FGFR inhibitor, infigratinib, has showed inhibitory effect on *FOP2-FGFR1* and *TPR-FGFR1 in vitro*.

To our knowledge, the fusion *KIF5B-FGFR1* has been only reported in a case of multicentric reticulohistiocytosis (MRH) (4). Our case is the first to identify the presence of this fusion gene in hematologic malignancies. Consistent with our patient, the MRH patient with KIF5B-FGFR1 carried no identifiable driver point mutations. Furthermore, bioinformatic analysis of RNA sequencing in Murakami et al. revealed that KIF5B-FGFR1 was associated with marked activated tyrosine kinase activity. Notably, the coiled-coil domain derived from KIF5B, which was contained by the fusion protein, could involve in protein dimerization, and then phosphorylate and constitutively activate the kinase domains domains (20). These findings suggest that the fusion protein might dimerize and activate FGFR1 tyrosine kinase to promote proliferation and survival of neoplasms without help from additional driver mutations, which needs to be further investigated in disease models.

In summary, we described an in-frame *KIF5B-FGFR1* fusion gene that consists of *KIF5B* exon 15 and *FGFR1* exon 10, which was firstly reported in hematologic malignancy. Similar to most EMS or *BCR-ABL1* like ALL cases, the patient suffered from disease relapse and then became refractory to traditional treatment. Future studies are needed to define the underlying mechanisms of this fusion gene on contributing to leukemogenesis, and new agents targeting the FGFR-related signaling pathway may provide a potential therapeutic option for hematopoietic neoplasms with *FGFR1* rearrangements. More importantly, RNA sequencing could improve detection rate of rare and novel fusion genes, and its future widespread application in clinical practice will provide a deeper understanding of disease biology.

## Data availability statement

The original contributions presented in the study are included in the article/**Supplementary Material**. Further inquiries can be directed to the corresponding authors.

## Ethics statement

The studies involving humans were approved by Ethics Committee of the First Affiliated Hospital of Soochow University. The studies were conducted in accordance with the local legislation and institutional requirements. The participants provided their written informed consent to participate in this study. Written informed consent was obtained from the individual(s) for the publication of any potentially identifiable images or data included in this article. All procedures performed in studies involving human participants were in accordance with the ethical standards of the First Affiliated Hospital of Soochow University committee and with the 1964 Helsinki declaration and its later amendments or comparable ethical standards. Informed consent was obtained from all individual participants included in the study.

## Author contributions

ZBZ: Writing – original draft, Formal Analysis, Visualization. YZ: Data curation, Writing – review & editing. ZW: Data curation, Writing – review & editing. ZZ: Validation, Writing – review & editing. LW: Methodology, Supervision, Writing – review & editing. LZ: Project administration, Validation, Writing – review & editing. SC: Funding acquisition, Project administration, Resources, Writing – review & editing.

## References

[1] Khoury JD Solary E Abla O Akkari Y Alaggio R Apperley JF. The 5th edition of the World Health Organization classification of haematolymphoid tumours: myeloid and histiocytic/dendritic neoplasms. Leukemia (2022) 36(7):1703–19. doi: 10.1038/s41375-022-01613-1 PMC9252913 35732831

[2] Arber DA Orazi A Hasserjian RP Borowitz MJ Calvo KR Kvasnicka HM. International Consensus Classification of Myeloid Neoplasms and Acute Leukemias: integrating morphologic, clinical, and genomic data. Blood (2022) 140(11):1200–28. doi: 10.1182/blood.2022015850 PMC9479031 35767897

[3] Li T Zhang G Zhang X Lin H Liu Q. The 8p11 myeloproliferative syndrome: Genotypic and phenotypic classification and targeted therapy. Front Oncol (2022) 12:1015792. doi: 10.3389/fonc.2022.1015792 36408177 PMC9669583

[4] Murakami N Sakai T Arai E Muramatsu H Ichikawa D Asai S. Targetable driver mutations in multicentric reticulohistiocytosis. Haematologica (2020) 105(2):e61–e4. doi: 10.3324/haematol.2019.218735 PMC7012471 31171640

[5] Tasian SK Loh ML Hunger SP. Philadelphia chromosome-like acute lymphoblastic leukemia. Blood (2017) 130(19):2064–72. doi: 10.1182/blood-2017-06-743252 PMC5680607 28972016

[6] Conant JL Czuchlewski DR. BCR-ABL1-like B-lymphoblastic leukemia/lymphoma: Review of the entity and detection methodologies. Int J Lab Hematol (2019) 41:126–30. doi: 10.1111/ijlh.13012 31069976

[7] Wang SA Orazi A Gotlib J Reiter A Tzankov A Hasserjian RP. The international consensus classification of eosinophilic disorders and systemic mastocytosis. Am J Hematol (2023) 98(8):1286–306. doi: 10.1002/ajh.26966 37283522

[8] Roberts KG Li Y Payne-Turner D Harvey RC Yang YL Pei D. Targetable kinase-activating lesions in ph-like acute lymphoblastic leukemia. N Engl J Med (2014) 371(11):1005–15. doi: 10.1056/NEJMoa1403088 PMC4191900 25207766

[9] Reshmi SC Harvey RC Roberts KG Stonerock E Smith A Jenkins H. Targetable kinase gene fusions in high-risk B-ALL: a study from the Children's Oncology Group. Blood (2017) 129(25):3352–61. doi: 10.1182/blood-2016-12-758979 PMC5482101 28408464

[10] Roberts KG Reshmi SC Harvey RC Chen IM Patel K Stonerock E. Genomic and outcome analyses of Ph-like ALL in NCI standard-risk patients: a report from the Children's Oncology Group. Blood (2018) 132(8):815–24. doi: 10.1182/blood-2018-04-841676 PMC6107876 29997224

[11] Paietta E Roberts KG Wang V Gu ZH Buck GAN Pei DQ. Molecular classification improves risk assessment in adult BCR-ABL1-negative B-ALL. Blood (2021) 138(11):948–58. doi: 10.1182/blood.2020010144 PMC9069478 33895809

[12] Jackson CC Medeiros LJ Miranda RN. 8p11 myeloproliferative syndrome: a review. Hum Pathol (2010) 41(4):461–76. doi: 10.1016/j.humpath.2009.11.003 20226962

[13] Urrea Pineda LY Perilla O Santiago-Pacheco V Trujillo Montoya S. Myeloproliferative syndrome with eosinophilia associated with translocation t(8; 13) and T-cell lymphoblastic lymphoma: A case report and review of the literature. Cureus (2022) 14(3):e22734. doi: 10.7759/cureus.22734 35386486 PMC8969320

[14] Montenegro-Garreaud X Miranda RN Reynolds A Tang G Wang SA Yabe M. Myeloproliferative neoplasms with t(8;22)(p11.2;q11.2)/BCR-FGFR1: a meta-analysis of 20 cases shows cytogenetic progression with B-lymphoid blast phase. Hum Pathol (2017) 65:147–56. doi: 10.1016/j.humpath.2017.05.008 28551329

[15] Chen MY Wang K Cai XH Zhang XW Chao HY Chen SN. Myeloid/lymphoid neoplasm with CEP110-FGFR1 fusion: An analysis of 16 cases show common features and poor prognosis. Hematology (2021) 26(1):153–9. doi: 10.1080/16078454.2020.1854493 33491601

[16] Umino K Fujiwara SI Ikeda T Toda Y Ito S Mashima K. Clinical outcomes of myeloid/lymphoid neoplasms with fibroblast growth factor receptor-1 (FGFR1) rearrangement. Hematology (2018) 23(8):470–7. doi: 10.1080/10245332.2018.1446279 29486661

[17] Konishi Y Kondo T Nakao K Asagoe K Otsuka Y Nishikori M. Allogeneic hematopoietic stem cell transplantation for 8p11 myeloproliferative syndrome with BCR-FGFR1 gene rearrangement: a case report and literature review. Bone Marrow Transplant (2019) 54(2):326–9. doi: 10.1038/s41409-018-0287-1 30087462

[18] Kreil S Adès L Bommer M Stegelmann F Ethell ME Lubking A. Limited efficacy of ponatinib in myeloproliferative neoplasms associated with FGFR1 fusion genes. Blood (2015) 126(23):2812. doi: 10.1182/blood.V126.23.2812.2812

[19] Gotlib J Kiladjian J-J Vannucchi A Rambaldi A Reiter A Shomali W. A phase 2 study of pemigatinib (FIGHT-203; INCB054828) in patients with myeloid/lymphoid neoplasms (MLNs) with fibroblast growth factor receptor 1 (FGFR1) rearrangement (MLN FGFR1). Blood (2021) 138(Supplement 1):385. doi: 10.1182/blood-2021-148103

[20] Takeuchi K Soda M Togashi Y Suzuki R Sakata S Hatano S. RET, ROS1 and ALK fusions in lung cancer. Nat Med (2012) 18(3):378–81. doi: 10.1038/nm.2658 22327623

