# Supplementary material for: Case Report: A novel FGFR1 fusion in acute B-lymphoblastic leukemia identified by RNA sequencing
Source: Front Oncol. 2023 Nov 1;13:1276695. doi: 10.3389/fonc.2023.1276695 (PMC10646441; doi:10.3389/fonc.2023.1276695)
Supplement: Supplementary file 1 [file Table_1.docx]

**Table S1. The next-generation sequencing (a panel of 172 genes)**

| ABL1 | BRAF | CD79B | CXCR4 | EZH2 | IKZF3 | MED12 | PDGFRA | RELN | STAG2 | TP63 |
| --- | --- | --- | --- | --- | --- | --- | --- | --- | --- | --- |
| ANKRD26 | BRCA1 | CDKN1A | DDX3X | FAM46C | IL7R | MEF2B | PDGFRB | RHOA | STAT1 | TP73 |
| APC | BRCA2 | CDKN2A | DDX41 | FAT1 | IRF4 | MPL | PHF6 | RPS15 | STAT2 | TRAF3 |
| ARID1A | BRCC3 | CDKN2B | DHX15 | FBXW7 | JAK1 | MUM1 | PIGA | RUNX1 | STAT3 | TYK2 |
| ARID1B | BTG1 | CDKN2C | DIS3 | FLT3 | JAK2 | MYC | PIK3CA | SAMHD1 | STAT4 | U2AF1 |
| ARID2 | BTK | CEBPA | DNM2 | FOXO1 | JAK3 | MYD88 | PIM1 | SETBP1 | STAT5A | UNC13D |
| ARID5B | CALR | CEBPE | DNMT3A | GATA1 | KDM6A | NF1 | PLCG1 | SETD2 | STAT5B | WT1 |
| ASXL1 | CARD11 | CHD8 | ECT2L | GATA2 | KIT | NFKB1 | PLCG2 | SF3B1 | STAT6 | XPO1 |
| ASXL2 | CBL | CIITA | EED | GATA3 | KLF2 | NFKB2 | PPM1D | SH2B3 | SUZ12 | ZAP70 |
| ATM | CCND1 | CRBN | EGFR | GNA13 | KMT2A | NFKBIE | PRDM1 | SMC1A | TBL1XR1 | ZBTB7A |
| B2M | CCND2 | CREBBP | EGR2 | HRAS | KMT2C | NOTCH1 | PTEN | SMC2 | TCF3 | ZMYM3 |
| BCL2 | CCND3 | CRLF2 | EP300 | ID3 | KMT2D | NOTCH2 | PTPN11 | SMC3 | TET1 | ZRSR2 |
| BCL6 | CD274 | CSF1R | EPOR | IDH1 | KRAS | NPM1 | PTPRD | SOS1 | TET2 |  |
| BCOR | CD28 | CSF3R | ERBB3 | IDH2 | MAP2K1 | NRAS | RAD21 | SPEN | TNFAIP3 |  |
| BCORL1 | CD58 | CSMD1 | ETNK1 | IGLL5 | MAPK1 | PAX5 | RAF1 | SRP72 | TNFRSF14 |  |
| BIRC3 | CD79A | CTCF | ETV6 | IKZF1 | MECOM | PDCD1LG2 | RB1 | SRSF2 | TP53 |  |
